# The paradox of moral salience: when ethical leadership amplifies the impact of coworker incivility on workplace cheating behavior

**DOI:** 10.3389/fpsyg.2026.1845596

**Published:** 2026-05-18

**Authors:** Baoyi Feng, Zhijun Chen

**Affiliations:** School of Management, Shandong University, Jinan, Shandong, China

**Keywords:** behavioral ethics, coworker incivility, ethical leadership, moral disengagement, workplace cheating behavior

## Abstract

**Purpose:**

Drawing upon moral disengagement theory, this study investigates the cognitive mechanisms and boundary conditions underlying how and when ambient coworker incivility spills over into workplace cheating behavior (WCB). Specifically, we offer a nuanced perspective on ethical leadership by examining its unintended amplifying effect in uncivil work environments.

**Methods:**

Utilizing a three-wave, time-lagged field survey with one-month intervals, we collected data from 252 full-time employees. We estimated a moderated mediation model using path analysis and bias-corrected bootstrapping to examine how ethical leadership conditionally shapes the indirect effect of coworker incivility on WCB via moral disengagement.

**Results:**

Results reveal that employee moral disengagement serves as a cognitive mechanism mediating the positive relationship between coworker incivility and subsequent cheating behavior. High ethical leadership amplifies the positive relationship between coworker incivility and moral disengagement. Consequently, the positive indirect effect of coworker incivility on WCB via moral disengagement is significantly stronger when ethical leadership is high rather than low.

**Conclusion:**

This study provides evidence that coworker incivility translates into workplace cheating by deactivating employees’ ethical self-regulation. Importantly, it highlights an unintended consequence of ethical leadership: when leaders espouse stringent moral standards but peer incivility remains unchecked, the stark contrast between normative expectations and interpersonal reality equips employees with potent cognitive justifications to morally disengage, thereby exacerbating ethical erosion.

## Introduction

Workplace cheating behavior (WCB), defined as “unethical acts intended to create an unfair advantage or avoid negative outcomes” (Mitchell et al., 2018), poses a pervasive and costly threat to organizational integrity and functioning. Consequently, organizational scholars have increasingly focused on identifying the workplace stressors that precipitate such self-serving misconduct. Among various stressors, coworker incivility, characterized as “low-intensity deviant behavior with ambiguous intent to harm” (Andersson and Pearson, 1999; Lim and Cortina, 2005), has emerged as a particularly ubiquitous and insidious interpersonal challenge. While extensive research demonstrates that targets of incivility often respond with negative emotions or direct retaliation against the instigator (e.g., tit-for-tat incivility; Andersson and Pearson, 1999), far less is known about how and why these everyday interpersonal slights spill over into unethical transgressions directed at the work itself, such as cheating. Addressing this theoretical puzzle is critical for understanding how seemingly interpersonal mistreatment can silently escalate into ethical breaches that compromise the broader organizational system.

To explicate this psychological spillover, we draw upon moral disengagement theory (Bandura et al., 1996; Bandura, 1999) to propose that employee moral disengagement serves as the critical cognitive mechanism linking exposure to coworker incivility to WCB. According to this theory, individuals typically refrain from unethical conduct because it violates internal moral standards and triggers self-censure. However, this self-regulatory system can be selectively deactivated through cognitive rationalizations, a process known as moral disengagement (Bandura et al., 1996). We argue that operating in an environment permeated by coworker incivility, where mutual disrespect and interpersonal norm violations are frequently exhibited among peers, provides employees with potent cognitive justifications to loosen their own moral boundaries.

Specifically, exposure to such ambient incivility leads employees to cognitively reframe the workplace as a context where ethical guidelines are malleable or unenforced. This perception facilitates cognitive strategies such as the diffusion of responsibility or advantageous comparison (Bandura, 1999; Moore et al., 2012), allowing individuals to rationalize that their own ethical compromises are acceptable given the broader climate of peer misconduct. Once moral self-regulation is deactivated, the psychological barriers to committing unethical acts are dismantled. Consequently, employees are more likely to engage in self-serving transgressions, such as WCB, because the moral cost of doing so has been cognitively neutralized by the prevailing uncivil context (Detert et al., 2008; Moore et al., 2012).

While the mediating role of moral disengagement elucidates how ambient incivility translates into cheating, it is equally critical to identify the boundary conditions under which this cognitive erosion is exacerbated. Traditionally, ethical leadership, defined as the demonstration and promotion of normatively appropriate conduct (Brown et al., 2005), has been predominantly viewed as a strong deterrent against workplace misconduct (Newman et al., 2020; Ogunfowora et al., 2022). However, we offer a more nuanced perspective by exploring the interactive tension between conflicting normative cues within the organizational context. Specifically, what happens when the stringent moral standards espoused by an ethical leader collide with a work environment permeated by peer incivility?

Drawing further on moral disengagement theory, we posit a counter-intuitive hypothesis: high ethical leadership paradoxically amplifies the positive relationship between ambient coworker incivility and employee moral disengagement. When an ethical leader explicitly advocates for respect yet ambient incivility remains rampant, it creates a stark cognitive discrepancy. Due to a strong contrast effect, this glaring inconsistency suggests that the leader’s ethical standards are merely rhetorical and unenforced (Dineen et al., 2006; Martin et al., 2014). Consequently, rather than buffering the negative effects of incivility, high ethical leadership inadvertently provides employees with even more potent cognitive justifications to disengage morally. Employees exposed to such contradictory cues are more likely to rely on specific cognitive rationalizations, such as displacement of responsibility or moral justification (Bandura, 1999; Ashforth and Anand, 2003). Thus, the presence of an ethical leader in a highly uncivil environment serves as a catalyst, rather than a deterrent, for moral disengagement.

**Figure 1:**
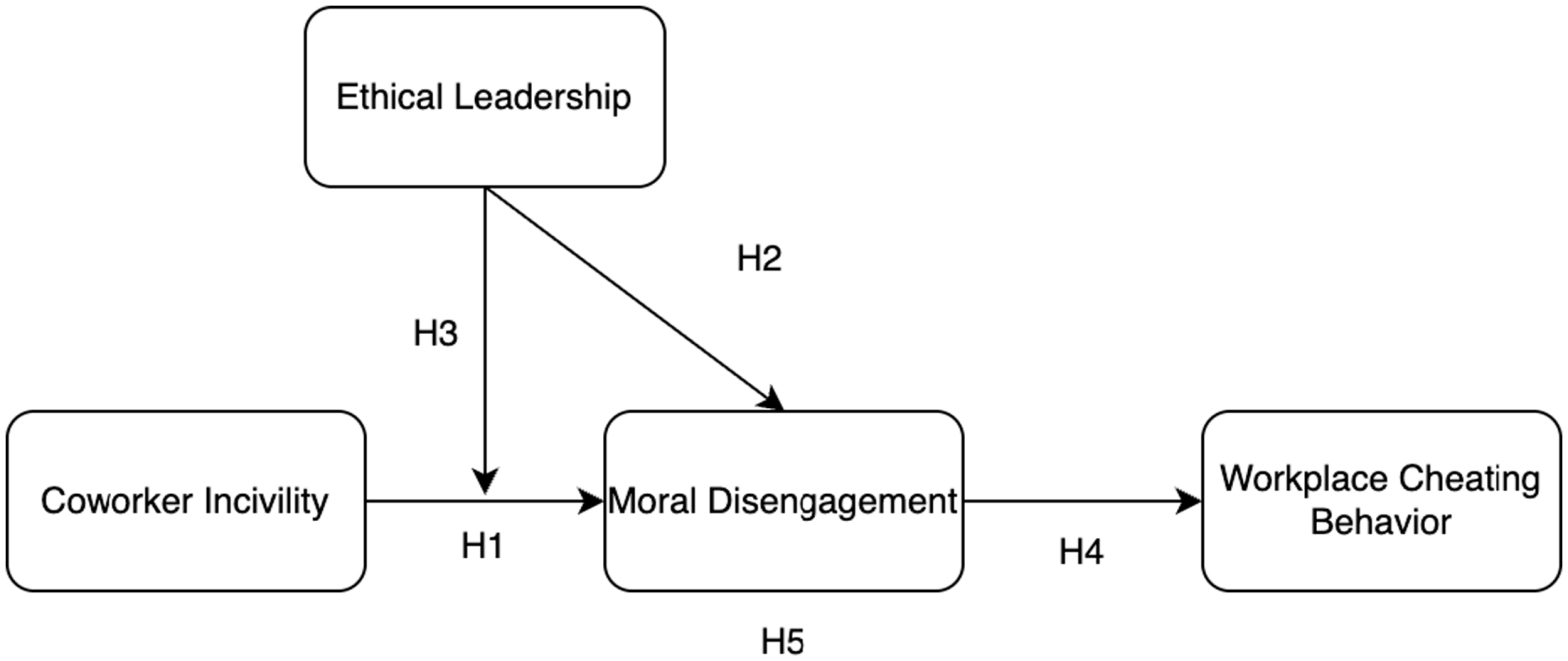
Hypothesized research model.

Taken together, the present study develops and tests a moderated mediation model to systematically examine the psychological mechanisms and boundary conditions underlying the translation of ambient coworker incivility into WCB. Specifically, we propose that employee moral disengagement mediates the positive relationship between exposure to coworker incivility and subsequent cheating behavior. Furthermore, we identify ethical leadership as a critical situational moderator in this process. We hypothesize that high levels of ethical leadership will amplify the positive effect of ambient incivility on moral disengagement, thereby strengthening the overall indirect effect on WCB. Figure 1 depicts our theoretical framework.

The present study advances the extant literature by unpacking the cognitive mechanisms that link ambient coworker incivility to organizational misconduct. By identifying moral disengagement as a critical mediator, we shift the theoretical focus toward the erosion of ethical self-regulation (Schilpzand et al., 2016). Furthermore, we offer a nuanced perspective on the traditional assumption that ethical leadership consistently deters misconduct (Brown et al., 2005). By highlighting the interactive tension between conflicting contextual cues (Newman et al., 2020), we reveal the unintended consequences of ethical leadership when it collides with a highly uncivil peer environment (Hayat et al., 2025). Ultimately, our model demonstrates how the discrepancy between interpersonal realities (i.e., ambient incivility) and normative expectations (i.e., ethical leadership) collectively shapes employees’ moral disengagement and subsequent cheating behaviors.

## Theoretical framework and hypotheses

### Coworker incivility and moral disengagement

According to moral disengagement theory (Bandura et al., 1996; Bandura, 1999), individuals typically regulate their behaviors to align with internalized moral standards, anticipating self-sanctions if they violate these norms. Meanwhile, individuals can cognitively deactivate these self-regulatory processes through a set of cognitive mechanisms, collectively termed moral disengagement, allowing them to act unethically without experiencing distress (Moore et al., 2012). We argue that observed coworker incivility provides the contextual impetus for such cognitive deactivation.

First, frequent exposure to rude and disrespectful treatments creates a perception of a hostile and norm-violating work environment. This toxic interpersonal context facilitates the displacement of responsibility (Bandura, 1999). When the workplace is permeated with incivility (Han et al., 2022; Magley et al., 2026), employees are more likely to attribute their own relaxation of moral standards to the broader dysfunctional environment or the instigators, rather than taking personal accountability. Consequently, employees may rationalize their relaxation of moral standards by perceiving that the widespread disregard for interpersonal rules by others diminishes their own obligation to strictly adhere to moral norms (Christian and Ellis, 2014).

Second, viewing the work environment as inherently unfair or hostile due to coworkers’ mistreatment, employees can cognitively justify their subsequent moral loosening as a legitimate defensive response or a deserved reaction against an unfair system (Hillebrandt and Barclay, 2013; Valle et al., 2019). Consequently, the pervasive experience of coworker incivility provides employees with convenient cognitive rationalizations to bypass their moral compass, leading to elevated levels of moral disengagement. Thus, we hypothesize the following:

*H1*: Coworker incivility is positively related to employee moral disengagement.

### Ethical leadership and moral disengagement

Leadership styles significantly shape employees’ moral cognition (Brown et al., 2005; Moore et al., 2019). Ethical leaders serve as salient moral role models in the workplace. By explicitly articulating ethical standards and holding employees accountable for their actions, ethical leaders cultivate a strong moral environment that reinforces employees’ internal moral self-regulatory systems (Bandura, 1999). This heightened moral awareness and strict adherence to ethical norms make it psychologically difficult for employees to utilize cognitive rationalizations, such as displacing responsibility or distorting consequences, to justify misconduct (Treviño et al., 2006; Hsieh et al., 2020). Consequently, the presence of ethical leadership effectively inhibits the cognitive deactivation process, thereby reducing employees’ tendency to morally disengage. Thus, we hypothesize the following:

*H2*: Ethical leadership is negatively related to employee moral disengagement.

### The moderating role of ethical leadership

Although ethical leadership generally inhibits baseline moral disengagement, we propose that it amplifies the positive relationship between coworker incivility and moral disengagement. Drawing on moral disengagement theory, we argue that the cognitive rationalizations required to deactivate moral self-regulation are highly contingent on the contextual cues provided by leaders and the broader social environment (Bandura et al., 1996; Bandura, 1999).

Under high ethical leadership, leaders explicitly promote moral standards and interpersonal respect, establishing high normative expectations. However, when ambient coworker incivility frequently occurs within this context, it triggers a sharp contrast effect, providing employees with highly potent cognitive justifications to morally disengage. Specifically, the stark cognitive discrepancy between the leader’s espoused expectations and the interpersonal reality facilitates the displacement of responsibility and the attribution of blame (Bandura et al., 1996; Christian and Ellis, 2014). Employees can cognitively rationalize their moral disengagement by blaming the leader’s failure to enforce the espoused rules (Greenbaum et al., 2015; Zoghbi-Manrique-de-Lara and Viera-Armas, 2019). They may justify their transgressions by reasoning that if heavily promoted ethical norms remain unenforced, they are absolved from the obligation to adhere to them. Ultimately, this salient inconsistency provides a powerful cognitive rationale for employees to bypass their moral compass.

Conversely, under low ethical leadership, the workplace lacks clear moral guidelines, and incivility may be perceived as a routine aspect of a morally ambiguous environment (Kish-Gephart et al., 2010). In such contexts, there is no salient ethical framework to “disengage” from. Because the environment does not explicitly demand high moral adherence, employees do not need to exert strong cognitive efforts, such as resolving a sharp expectation-reality discrepancy or displacing responsibility onto the leader, to rationalize their own moral relaxation. Consequently, the cognitive mechanisms of moral disengagement are less intensely activated by coworker incivility when ethical leadership is low. Thus, we hypothesize the following:

*H3*: Ethical leadership moderates the positive relationship between coworker incivility and employee moral disengagement. Specifically, the positive relationship is stronger when ethical leadership is high rather than low.

### Moral disengagement and WCB

Extensive research drawing on moral disengagement theory has established a robust link between moral disengagement and various forms of unethical conduct (Detert et al., 2008; Moore et al., 2012). According to Bandura (1999), when individuals successfully deactivate their internal moral self-regulatory processes, they are freed from the anticipatory guilt and self-censure that typically deter wrongdoing. Consequently, morally disengaged employees are more likely to engage in WCB. Because they have cognitively reframed these transgressions as acceptable, justified, or consequence-free, the psychological barriers to committing such acts are removed (Newman et al., 2020; Ogunfowora et al., 2022). Therefore, consistent with prior literature, we expect a positive relationship between moral disengagement and WCB.

*H4*: Employee moral disengagement is positively related to WCB.

Taking the rationales for Hypothesis 3 and Hypothesis 4 together, we further propose a moderated mediation model.

*H5*: Ethical leadership moderates the indirect effect of coworker incivility on WCB via moral disengagement. Specifically, the positive indirect effect is stronger when ethical leadership is high rather than low.

## Methods

### Participants and procedure

Data were collected from full-time employees working at a large construction company located in central China using a convenience sampling method. We purposefully selected this industry because its high-pressure, project-based environment makes it highly relevant for examining interpersonal stressors like workplace incivility and leadership dynamics. With the assistance of the human resources department, we distributed questionnaires to 320 employees. To encourage participation, respondents received a cash reward of 10 RMB for completing each wave, totaling 30 RMB (approximately $4.4 USD). To match the surveys across the three waves while ensuring confidentiality, participants were required to input the last six digits of their mobile phone numbers as unique identification codes, which protected their anonymity.

To minimize potential common method variance (Podsakoff et al., 2012) and establish temporal precedence for our hypothesized model, we conducted a three-wave field survey with a one-month time lag between each wave. At Time 1, participants reported their demographic information (i.e., gender, age, and education), coworker incivility, and ethical leadership. One month later (Time 2), participants were asked to rate their moral disengagement. After another month (Time 3), participants reported their WCB.

After matching the data across all three time points and excluding incomplete responses or those that failed the attention check, we obtained a final valid sample of 252 employees, yielding an effective response rate of 78.75%. Among the final 252 participants, 70.6% were male. The average age of the participants was 30.37 years (SD = 13.77). Regarding educational background, 27.8% held a high school diploma or below, 51.2% held an associate degree, and 21.0% held a bachelor’s degree.

### Measures

Because the original measurement scales were developed in English, we followed the standard back-translation procedure (Brislin, 1980) to create the Chinese versions of the survey instruments. Unless otherwise specified, participants responded to all items on a 5-point Likert scale ranging from 1 (strongly disagree) to 5 (strongly agree).

Coworker incivility. We measured coworker incivility at Time 1 using a 4-item scale adapted from Lim and Cortina (2005). Participants were asked to indicate the frequency with which they experienced uncivil behaviors from their coworkers. A sample item is “My coworker put others down or was condescending to others.” The Cronbach’s *α* for this scale was 0.87.

Ethical leadership. At Time 1, employees rated their supervisors’ ethical leadership using the 10-item scale developed by Brown et al. (2005). A sample item is “My leader can be trusted.” The Cronbach’s α for this scale was 0.90.

Moral disengagement. At Time 2, we assessed employees’ moral disengagement using a 4-item scale validated by Welsh et al. (2020). A sample item is “Questionable behavior is sometimes justifiable given the actual interpersonal atmosphere in my organization.” The Cronbach’s α for this scale was 0.83.

WCB. At Time 3, employees self-reported their WCB using a 7-item scale developed by Mitchell et al. (2018). A sample item is “I made it look like I am working when I am not.” The Cronbach’s α for this scale was 0.84.

Control variables. Consistent with prior research on moral literature (e.g., Allmon et al., 2000; Kish-Gephart et al., 2010), we controlled for employees’ gender, age, and education level in our analyses, as these demographic characteristics may potentially influence individuals’ moral disengagement and subsequent WCB.

### Data analysis

We conducted our statistical analyses using R software with the *lavaan* package (Rosseel, 2012). Our analytical procedures proceeded in three main steps. First, to evaluate the discriminant validity of our focal constructs, we performed confirmatory factor analyses. Following established methodological recommendations (Hu and Bentler, 1999; Kline, 2016), model fit was assessed using a combination of absolute and incremental fit indices, including the comparative fit index (CFI), Tucker-Lewis index (TLI), root mean square error of approximation (RMSEA), and standardized root mean square residual (SRMR). Generally, CFI and TLI values greater than 0.90, and RMSEA and SRMR values below 0.08 indicate an acceptable model fit (Kline, 2016).

Second, we employed path analysis to test the main and moderation hypotheses. To minimize potential multicollinearity issues and facilitate the interpretation of the interaction term, the independent variable (coworker incivility) and the moderator (ethical leadership) were mean-centered prior to creating the interaction term (Aiken and West, 1991). For significant interactions, we conducted simple slope analyses and plotted the effects at one standard deviation above and below the mean of the moderator, as recommended by Aiken and West (1991).

Finally, to test the moderated mediation model, we calculated the index of moderated mediation (Hayes, 2015). We utilized bias-corrected bootstrapping with 5,000 resamples to estimate the 95% confidence intervals for the conditional indirect effects. Bootstrapping is highly recommended for testing indirect effects because it avoids the assumption of a normal sampling distribution and provides greater statistical power (Preacher et al., 2007).

## Results

### Confirmatory factor analyses

To examine the discriminant validity of our focal constructs (i.e., coworker incivility, ethical leadership, moral disengagement, and WCB), we conducted confirmatory factor analyses. The hypothesized 4-factor model demonstrated an acceptable fit to the data: *χ*^2^ (269) = 512.70, CFI = 0.92, TLI = 0.91, RMSEA = 0.06, SRMR = 0.06. Furthermore, we compared the hypothesized 4-factor model with all 6 alternative 3-factor models (by combining different pairs of constructs). Results revealed that all alternative models yielded significantly poorer fit, with *χ*^2^ (272) ranging from 843.49 to 1209.48. Chi-square difference tests indicated that the hypothesized 4-factor model fit the data significantly better than all alternative 3-factor models [Δ*χ*^2^ (3) ranging from 330.79 to 696.78, *p* < 0.001]. Together, these results provide strong support for the discriminant validity of our focal variables.

### Hypothesis tests

In Table 1, we presented the descriptive statistics and correlations for all variables included in our study. We then utilized path analysis to test our hypotheses, and results are presented in Table 2. Hypothesis 1 proposed that coworker incivility is positively related to employee moral disengagement. As shown in Model 1 of Table 2, after accounting for the control variables, the unstandardized path coefficient from coworker incivility to moral disengagement was positive and statistically significant (*b* = 0.28, *SE* = 0.07, *p* < 0.01). Thus, Hypothesis 1 was supported.

**Table 1:** Descriptive statistics and correlations.

Variable	Mean	SD	1	2	3	4	5	6	7
1. Gender	1.29	0.46							
2. Age	30.37	13.77	−0.06						
3. Education	1.93	0.70	0.13*	−0.31**					
4. Coworker Incivility	2.24	0.75	−0.03	0.06	−0.13*	(0.87)			
5. Ethical Leadership	4.20	0.53	−0.00	−0.01	0.05	−0.32**	(0.90)		
6. MD	2.46	0.79	−0.08	0.07	−0.12	0.28**	−0.15*	(0.83)	
7. WCB	2.31	0.60	−0.00	−0.07	0.04	0.24**	−0.13*	0.29**	(0.84)

*N* = 252. MD, Moral Disengagement; WCB, Workplace Cheating Behavior. Gender was coded as 1 = male, 2 = female. Education was coded as 1 = high school and below, 2 = associate degree, 3 = bachelor’s degree, 4 = graduate degree and above. Cronbach’s αs are reported in parentheses along the diagonal. ∗*p* < 0.05; ∗∗*p* < 0.01.

**Table 2:** Results of path analysis.

Variables	Model 1		Model 2		Model 3	
	MD	WCB	MD	WCB	MD	WCB
Intercept	2.69**(0.23)	1.81**(0.21)	2.73**(0.24)	1.77**(0.22)	2.77**(0.22)	1.84**(0.22)
Gender	−0.11(0.11)	0.01(0.08)	−0.11(0.11)	0.01(0.08)	−0.14(0.10)	0.01(0.08)
Age	0.00(0.00)	0.00(0.00)	0.00(0.00)	0.00(0.00)	0.00(0.00)	0.00(0.00)
Education	−0.07(0.07)	0.06(0.06)	−0.10(0.07)	0.05(0.06)	−0.07(0.07)	0.06(0.06)
Coworker Incivility	0.28**(0.07)	0.15**(0.05)			0.20**(0.07)	−0.06(0.08)
Ethical Leadership			−0.21*(0.09)	−0.11(0.07)	−0.13(0.09)	0.14*(0.06)
Interaction					0.31**(0.11)	0.03(0.09)
MD		0.19**(0.05)		0.22**(0.05)		0.19**(0.05)
*R* ^2^	0.09	0.12	0.04	0.10	0.12	0.13

*N* = 252. MD, Moral Disengagement; WCB, Workplace Cheating Behavior; Interaction, Coworker Incivility × Ethical Leadership. Unstandardized path coefficients are reported, with standard errors in parentheses. Predictors were mean-centered prior to forming the interaction term. *p < 0.05; **p < 0.01.

Hypothesis 2 predicted that ethical leadership is negatively related to employee moral disengagement. Consistent with our expectation, results in Model 2 of Table 2 revealed a significant negative relationship between ethical leadership and moral disengagement (*b* = −0.21, *SE* = 0.09, *p* < 0.05). Therefore, Hypothesis 2 received support.

**Figure 2:**
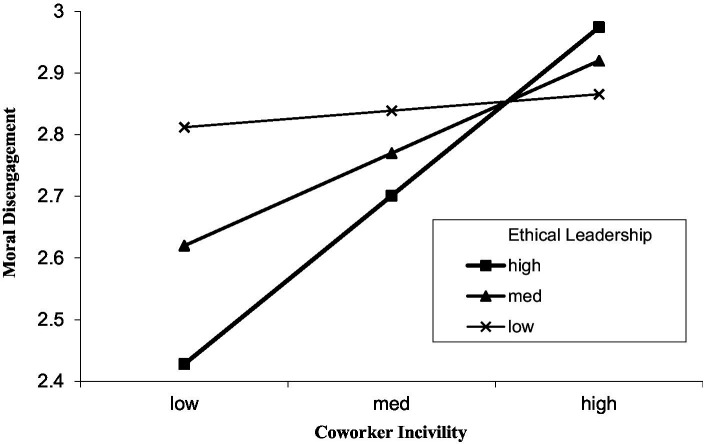
Moderating effect of ethical leadership on relationship between coworker incivility and moral disengagement.

Hypothesis 3 proposed that ethical leadership moderates the positive relationship between coworker incivility and employee moral disengagement, such that the relationship is stronger when ethical leadership is high. As shown in Model 3 of Table 2, the interaction term between coworker incivility and ethical leadership was significantly and positively related to moral disengagement (*b* = 0.31, *SE* = 0.11, *p* < 0.01). To further interpret the nature of this significant interaction, we conducted simple slope analyses and plotted the interaction effect at high (+1 SD) and low (−1 SD) levels of ethical leadership (see Figure 2). The simple slope tests revealed that the positive relationship between coworker incivility and moral disengagement was strong and significant when ethical leadership was high (*b* = 0.36, *SE* = 0.09, *p* < 0.01), but became weak and non-significant when ethical leadership was low (*b* = 0.03, *SE* = 0.09, *ns*). These results indicate that high ethical leadership exacerbates the positive effect of coworker incivility on moral disengagement. Thus, Hypothesis 3 was supported.

Hypothesis 4 predicted that employee moral disengagement is positively related to WCB. As shown in the WCB column of Model 3 in Table 2, after controlling for demographic variables and the independent variables, moral disengagement was positively and significantly related to WCB (*b* = 0.19, *SE* = 0.05, *p* < 0.01). Therefore, Hypothesis 4 was supported.

Hypothesis 5 proposed a moderated mediation model, such that ethical leadership moderates the positive indirect effect of coworker incivility on WCB via moral disengagement. To test this hypothesis, we estimated the conditional indirect effects and the index of moderated mediation using bias-corrected bootstrapping with 5,000 resamples.

As presented in Table 3, the index of moderated mediation was significant [*b* = 0.06, *SE* = 0.03, 95% CI (0.01, 0.11)], as the confidence interval excluded zero. Specifically, the indirect effect of coworker incivility on WCB via moral disengagement was positive and significant when ethical leadership was high [+1 SD; *b* = 0.07, *SE* = 0.02, 95% CI (0.02, 0.12)], but was not significant when ethical leadership was low [−1 SD; *b* = 0.01, *SE* = 0.02, 95% CI (−0.03, 0.04)]. These results demonstrate that the positive indirect effect is stronger under high ethical leadership. Thus, Hypothesis 5 was supported.

**Table 3:** Results of moderated mediation effect.

Conditions	*b*	SE	95% CI
Moderated mediation effect	0.06*	0.03	[0.01, 0.11]
High EL (+1 SD)	0.07*	0.02	[0.02, 0.12]
Low EL (−1 SD)	0.01	0.02	[−0.03, 0.04]

*N* = 252. EL, Ethical Leadership; CI, Confidence interval. Unstandardized coefficients (b) are reported. The moderated mediation effect represents the index of moderated mediation. The 95% CIs were computed using bias-corrected bootstrapping with 5,000 resamples. *p < 0.05.

## Discussion

The primary objective of this research was to delineate how and when everyday interpersonal mistreatment, specifically, ambient coworker incivility, escalates into self-serving organizational transgressions. Drawing on moral disengagement theory, our results confirm that moral disengagement acts as a crucial cognitive conduit linking exposure to uncivil peers with WCB. By navigating a disrespectful environment, employees cognitively deactivate their ethical self-regulation, which subsequently removes the psychological barriers to cheating. Most intriguingly, our study uncovers a paradoxical boundary condition that adds nuance to the traditional view of ethical leadership as a consistent deterrent against misconduct. We found that high levels of ethical leadership actually exacerbate the positive relationship between ambient incivility and moral disengagement, thereby strengthening the indirect effect on cheating. Ultimately, these findings suggest that when a leader’s strict moral rhetoric collides with an unchecked, uncivil peer environment, the resulting normative discrepancy inadvertently equips employees with powerful cognitive rationalizations to bypass their moral compass.

### Theoretical implications

Our first theoretical implication lies in extending the literature on the spillover effects of workplace incivility by illuminating the cognitive mechanisms that link interpersonal mistreatment to organizational misconduct. While a substantial body of research has established that exposure to incivility typically elicits affective reactions, withdrawal, or direct retaliation against the instigator (Andersson and Pearson, 1999; Cortina et al., 2001; Porath and Erez, 2007), far less is known about how these everyday interpersonal slights escalate into self-serving ethical transgressions, such as cheating (Mitchell et al., 2018). By identifying moral disengagement (Bandura, 1999; Moore et al., 2012) as the critical cognitive bridge, we shift the theoretical narrative from an emotion-driven or retaliatory paradigm (Hayat et al., 2025) to one of cognitive erosion. We demonstrate that a work environment permeated by peer disrespect does more than just foster negative feelings; it fundamentally alters employees’ moral cognition. Ambient incivility signals that normative boundaries are malleable, thereby equipping employees with convenient cognitive rationalizations to deactivate their ethical self-regulation (Detert et al., 2008). Consequently, our findings provide a more nuanced understanding of the insidious nature of incivility: it silently dismantles the psychological barriers to wrongdoing, allowing employees to engage in cheating behaviors while remaining insulated from the self-censure that typically deters such acts.

Second, our findings offer a counter-narrative to the dominant view that ethical leadership universally serves as a robust buffer against workplace misconduct (Al Halbusi et al., 2025; Brown et al., 2005; Jeong et al., 2025; Kalra et al., 2025; Mayer et al., 2012; Ogunfowora et al., 2022; Qasim and Laghari, 2025). By revealing that high ethical leadership paradoxically amplifies the positive relationship between ambient incivility and moral disengagement, we illuminate a critical blind spot in the ethical leadership literature. When a leader explicitly espouses high moral standards but ambient incivility remains unchecked among peers, it creates a stark normative discrepancy. Rather than mitigating unethical tendencies, this glaring contradiction provides employees with highly potent cognitive justifications, such as the displacement of responsibility (Bandura, 1999), to morally disengage. Employees can easily rationalize their own ethical compromises by blaming the leader’s failure to enforce the promoted norms, a reaction to low behavioral integrity (Simons, 2002). Consequently, this study enriches the boundary conditions of ethical leadership by demonstrating that leadership effectiveness cannot be fully understood in isolation from the surrounding peer environment (Salancik and Pfeffer, 1978; Oc, 2018). Espousing ethical values without corresponding enforcement in a toxic context may inadvertently accelerate, rather than arrest, ethical erosion.

Finally, our research advances moral disengagement theory by highlighting the interactive tension between conflicting contextual cues (Fida et al., 2025). While prior literature has predominantly examined the antecedents of moral disengagement in isolation, focusing either on leadership styles or peer behaviors as independent triggers, our study paints a more complex, interactive picture. We demonstrate that the deactivation of moral self-regulation is not merely a straightforward reaction to a single stressor. Instead, it is a sophisticated cognitive response to the glaring discrepancy between interpersonal realities (i.e., coworker incivility) and normative expectations (i.e., ethical leadership). By conceptualizing moral disengagement as a product of contextual dissonance, we answer calls for a more integrative approach to ethical decision-making (Tenbrunsel and Smith-Crowe, 2008; Treviño et al., 2014). Employees do not process leadership rhetoric and peer behavior in a vacuum; rather, they actively compare and synthesize these social cues (Salancik and Pfeffer, 1978). When these organizational signals sharply collide, the resulting dissonance provides the exact fertile psychological ground needed for specific moral disengagement mechanisms, such as moral justification and displacement of responsibility (Bandura, 1999), to flourish. Thus, our moderated mediation model provides a more holistic and dynamic understanding of how complex, and sometimes contradictory, organizational environments collectively shape employees’ moral cognition.

### Practical implications

Our findings yield several imperative insights for managerial practice, particularly regarding the unintended consequences of ethical leadership in uncivil environments. First, it is crucial for leaders to consistently align their rhetoric with tangible actions to avoid creating a stark contrast between ethical expectations and workplace reality. Our counter-intuitive finding, that ethical leadership may exacerbate misconduct when paired with high ambient incivility, serves as a cautionary note regarding moral advocacy. When managers espouse lofty ethical standards without enacting substantive disciplinary measures against uncivil behaviors, such advocacy risks backfiring, potentially fostering cynicism and facilitating moral disengagement. Thus, to be truly effective, ethical leadership should ideally be accompanied by consistent behavioral enforcement.

Second, organizations are encouraged to cultivate a climate that actively discourages ambient incivility. While managers may be tempted to dismiss minor interpersonal frictions as trivial or private matters, our results underscore that these everyday slights can act as a catalyst for cognitive erosion, gradually undermining the collective moral compass and ultimately escalating into organizational transgressions such as cheating. Finally, organizations can benefit from proactively monitoring and addressing employees’ moral cognitive dynamics. Through targeted ethics training and interventions, management can equip employees to identify and challenge common moral disengagement rationalizations, such as displacing blame onto leaders’ inaction or diffusing responsibility among uncivil peers, thereby interrupting the cognitive pathways that lead to misconduct.

### Limitations and future directions

Despite its theoretical and practical implications, this study has several limitations that offer fruitful avenues for future research. First, although we utilized a time-lagged design to mitigate common method variance, the correlational nature of our data precludes definitive causal inferences. Future research could employ experimental designs or experience sampling methods (ESM) to capture the dynamic, within-person fluctuations of moral disengagement and establish stricter causality.

Second, because our data were collected exclusively in China, the cross-cultural generalizability of our findings remains to be tested. Cultural dimensions like high power distance may uniquely amplify employees’ sensitivity to the discrepancy between leadership rhetoric and peer incivility. Future research could cross-validate our model in Western or other diverse cultural contexts to establish the boundary conditions of this paradoxical effect.

## Conclusion

In conclusion, this study unveils the insidious cognitive pathway through which ambient workplace incivility spills over into self-serving cheating behavior. More importantly, it highlights the unintended consequences of ethical leadership. By demonstrating that high ethical advocacy, when juxtaposed with unchecked peer incivility, paradoxically exacerbates employees’ moral disengagement, we add nuance to the assumption that ethical leadership acts as a consistent deterrent against misconduct. Ultimately, our findings serve as a potent reminder that in the absence of rigorous behavioral enforcement, moral rhetoric may inadvertently cultivate the very unethical behaviors it seeks to prevent, transforming a well-intentioned ethical climate into a contradictory environment that fosters moral disengagement.

## Data Availability

The raw data supporting the conclusions of this article will be made available by the authors, without undue reservation.
